# Spironolactone-Induced Bilateral Gynecomastia in a Patient With Comorbid Conditions

**DOI:** 10.7759/cureus.114587

**Published:** 2026-08-16

**Authors:** Shruti Vihang Brahmbhatt, Deshna Jain

**Affiliations:** 1 Department of Pharmacology, Shrimati Bhikhiben Kanjibhai Shah Medical Institute and Research Centre, Sumandeep Vidyapeeth (Deemed to be University), Vadodara, IND

**Keywords:** adverse drug reaction, bilateral breast swelling, chronic liver disease, drug-induced gynecomastia, hormonal imbalance, spironolactone, type 2 diabetes mellitus

## Abstract

Gynecomastia is a benign proliferation of male breast glandular tissue caused by an imbalance between estrogen and androgen activity. Spironolactone, a potassium-sparing diuretic commonly prescribed for edema and ascites in chronic liver disease, is a recognized cause of drug-induced gynecomastia because of its antiandrogenic effects. We report the case of a man in his 50s with chronic liver disease and type 2 diabetes mellitus who presented with bilateral breast swelling and mild tenderness of one month’s duration. Clinical examination revealed bilateral soft glandular enlargement without nipple discharge, a hard mass, or axillary lymphadenopathy. Breast ultrasonography demonstrated diffuse fibroglandular proliferation with subareolar thickening consistent with bilateral gynecomastia. Laboratory investigations showed mildly reduced serum testosterone with relative estrogen predominance, while prolactin, luteinizing hormone, follicle-stimulating hormone, and thyroid function tests were within normal limits. Chronic liver disease likely contributed to hormonal imbalance through impaired estrogen metabolism and altered sex hormone regulation, thereby increasing susceptibility to spironolactone-induced gynecomastia. Spironolactone therapy was discontinued, and conservative management with clinical monitoring was advised. The patient showed gradual symptomatic improvement during follow-up. This case highlights the importance of recognizing medication-induced endocrine adverse effects, particularly in patients with chronic systemic illnesses such as hepatic dysfunction.

## Introduction

Gynecomastia refers to the benign enlargement of male breast glandular tissue resulting from an imbalance between estrogen and androgen activity [1]. It may occur physiologically or secondary to systemic disorders, endocrine abnormalities, chronic diseases, or medications [2]. Spironolactone-induced gynecomastia is a well-recognized and dose-dependent adverse effect, with reported incidence rates ranging from approximately 5-10% in routine clinical use and increasing with higher daily doses and prolonged duration of therapy. Chronic liver disease is an important predisposing factor because impaired hepatic metabolism alters estrogen clearance, increases peripheral aromatization of androgens to estrogens, elevates sex hormone-binding globulin levels, and reduces free testosterone concentrations, thereby promoting estrogen predominance and glandular breast tissue proliferation [3].

Spironolactone is a potassium-sparing diuretic frequently prescribed for edema and ascites associated with chronic liver disease. However, because of its antiandrogenic properties, including androgen receptor blockade and inhibition of testosterone synthesis, spironolactone is a recognized cause of drug-induced gynecomastia. Chronic liver disease impairs the hepatic metabolism of estrogens, leading to elevated circulating estrogen levels and reduced bioavailable testosterone. Spironolactone further exacerbates this hormonal imbalance through its antiandrogenic properties, including competitive inhibition of androgen receptors, suppression of testosterone synthesis, and enhanced peripheral conversion of testosterone to estradiol [4]. Consequently, the combined effects of liver disease and spironolactone shift the estrogen-androgen balance toward estrogenic predominance, promoting proliferation of breast glandular tissue and increasing the risk of gynecomastia. Early identification of medication-induced gynecomastia is important to prevent unnecessary investigations, patient anxiety, and inappropriate interventions.

While spironolactone-associated gynecomastia is a well-known adverse drug reaction, it can be difficult to diagnose in patients with chronic liver disease because both the underlying illness and spironolactone therapy may cause hormonal imbalance and breast growth. The purpose of this case study is to demonstrate how a systemic illness and its treatment may contribute to an endocrine adverse effect in an additive or overlapping manner. This highlights the significance of carefully evaluating comorbid conditions and medication history for proper clinical attribution and management.

## Case presentation

A man in his 50s who was working as a driver presented in May 2024 with bilateral breast swelling of one month’s duration, accompanied by mild tenderness on palpation. The swelling had a gradual onset. He also reported generalized weakness for one to three months and bilateral pedal edema for four to five months. There was no history of fever, cough, dyspnea, chest pain, abdominal pain, nausea, vomiting, nipple discharge, trauma, or weight loss.

He had a history of type 2 diabetes mellitus for two years and was taking metformin 500 mg and glimepiride 2 mg every 12 hours. The patient also had a known history of chronic liver disease associated with pedal edema for two years. Initially, he received spironolactone 100 mg and torsemide 10 mg once daily for six months, after which the spironolactone dose was tapered to 50 mg, while torsemide 10 mg was continued. He subsequently developed gradual bilateral breast enlargement associated with mild tenderness, which had been present for one month before seeking medical attention. Clinical examination, hormonal evaluation, and breast ultrasonography supported the diagnosis of gynecomastia, while other potential causes, including malignancy and endocrine disorders, were considered less likely. In view of the known association between spironolactone and gynecomastia, spironolactone was discontinued. At follow-up, the patient reported improvement in breast tenderness and no further progression of breast enlargement, supporting a possible causal relationship between spironolactone therapy and the adverse event.

**Figure 1:**
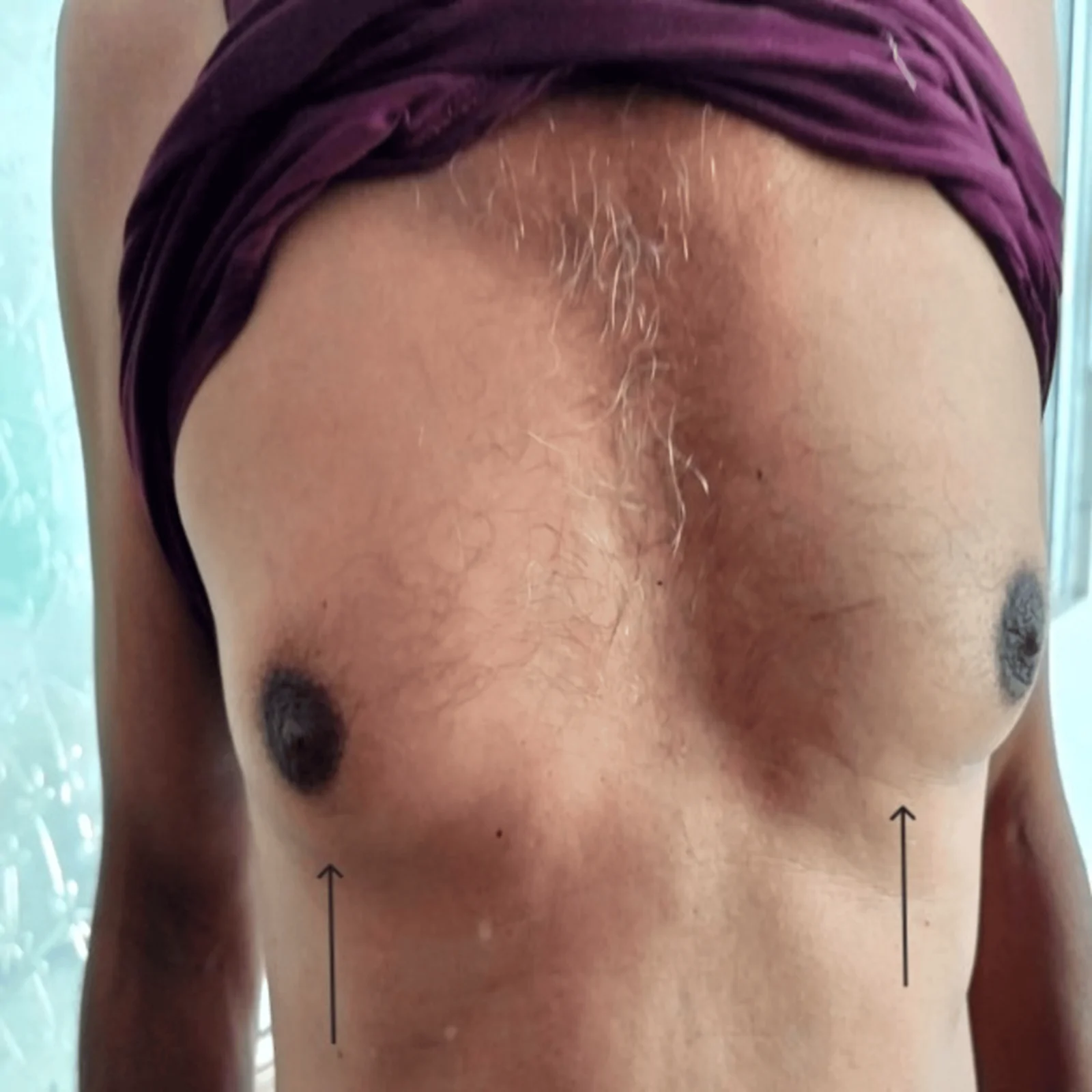
Clinical photograph showing bilateral breast enlargement consistent with gynecomastia.

On general examination, the patient was afebrile and hemodynamically stable. He was conscious, oriented, and comfortable. Bilateral pitting edema, which was more prominent on the left side, was noted. Breast examination revealed bilateral soft glandular enlargement with mild tenderness (Figure 1). No hard lumps, nipple discharge, skin retraction, or significant axillary lymphadenopathy were observed.

Investigation

Routine hematological and biochemical investigations were performed along with glycemic monitoring. The complete blood count was within normal limits. Liver function tests revealed hypoalbuminemia with mildly elevated serum bilirubin and liver enzyme levels, consistent with chronic liver disease. Renal function tests and serum electrolytes were within acceptable limits. Fasting and postprandial blood glucose levels were elevated, consistent with underlying type 2 diabetes mellitus.

Laboratory investigations revealed mildly elevated aspartate aminotransferase levels (48 IU/L), reduced total protein (5.8 g/dL), a low albumin-to-globulin ratio (1.15), and hypokalemia (3.2 mmol/L), findings consistent with the patient’s underlying chronic liver disease. Hormonal evaluation was performed to exclude other endocrine causes of gynecomastia. Serum testosterone was mildly reduced (245 ng/dL; reference range, 300-1000 ng/dL), while serum estradiol was elevated (58 pg/mL; reference range, 10-40 pg/mL). Serum luteinizing hormone, follicle-stimulating hormone, prolactin, and thyroid function tests were within normal limits. These findings supported a hormonal imbalance potentially related to chronic liver disease and spironolactone therapy (Table 1).

**Table 1:** Key laboratory investigations at presentation. Key biochemical and hormonal investigations demonstrating underlying chronic liver disease and hormonal imbalance associated with gynecomastia.

Parameter	Result	Reference range
Hemoglobin A1c	8.30%	4-6%
Aspartate aminotransferase	48 IU/L	<40 IU/L
Alanine aminotransferase	31 IU/L	<40 IU/L
Total bilirubin	0.7 mg/dL	0.2-1.0 mg/dL
Total protein	5.8 g/dL	6-8 g/dL
Serum albumin	3.1 g/dL	2.7-5.0 g/dL
Serum potassium	3.2 mmol/L	3.5-5.5 mmol/L
Serum testosterone	245 ng/dL	300-1000 ng/dL
Serum estradiol	58 pg/mL	10-40 pg/mL

**Figure 2:**
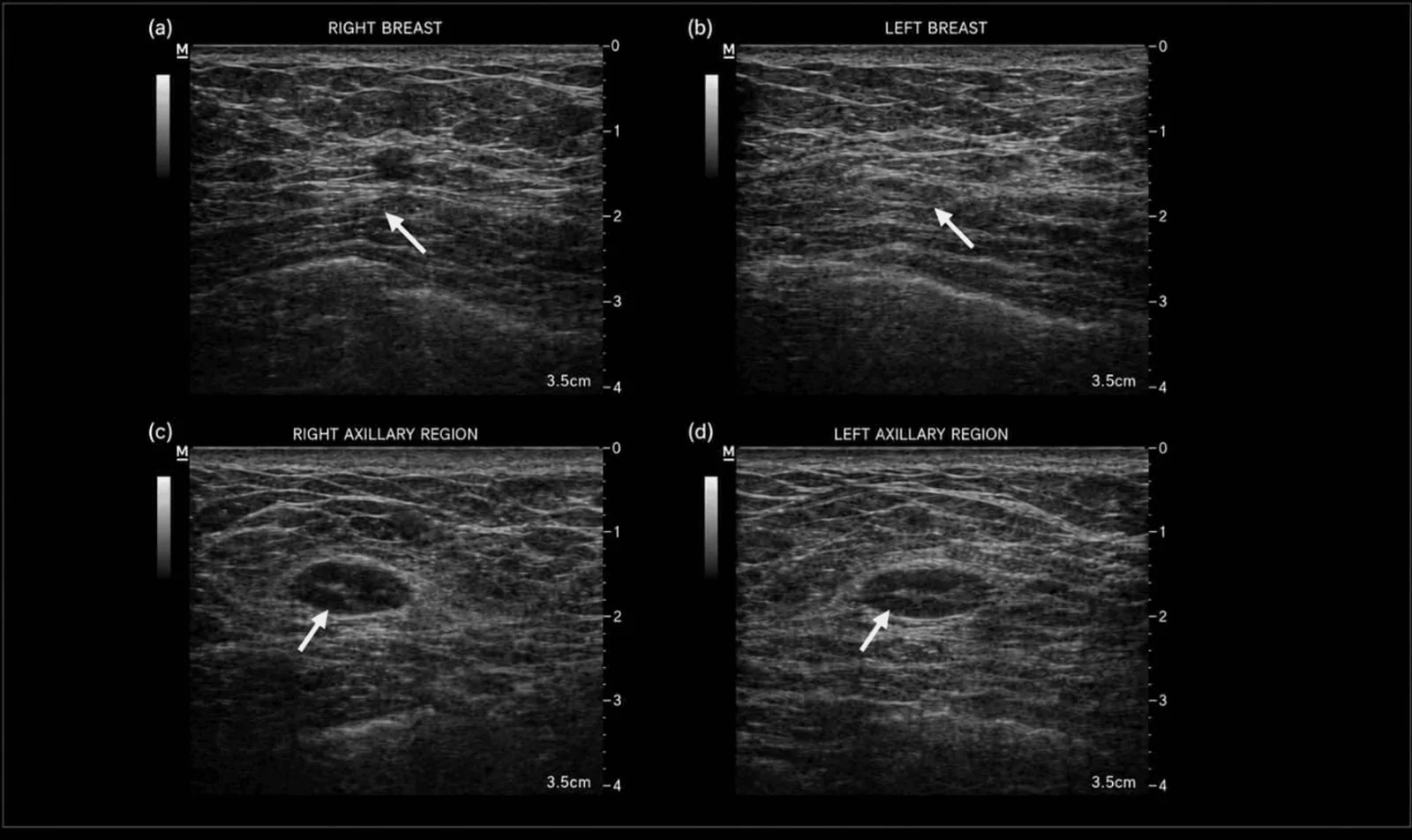
Ultrasonographic appearance of bilateral gynecomastia showing subareolar fibroglandular proliferation. (a) Right breast ultrasound showing diffuse fibroglandular parenchymal tissue involving the subareolar region with maximum thickness measuring 1.3 cm (arrow). (b) Left breast ultrasound showing diffuse fibroglandular parenchymal tissue involving the subareolar region with maximum thickness measuring 1.25 cm (arrow). (c) Right axillary ultrasound showing a subcentimetric lymph node (arrow). (d) Left axillary ultrasound showing a subcentimetric lymph node (arrow).

Breast ultrasonography demonstrated diffuse fibroglandular parenchyma with subareolar thickening measuring approximately 1.3 cm on the right and 1.25 cm on the left. Duct ectasia was observed without focal mass lesions. A subcentimetric, morphologically normal axillary lymph node was identified. These findings were consistent with bilateral gynecomastia (Figure 2).

Differential diagnosis

Differential diagnoses included male breast carcinoma, pseudogynecomastia, endocrine disorders, drug-induced gynecomastia, and gynecomastia secondary to chronic liver disease. Malignancy was considered unlikely because of bilateral symmetrical enlargement, absence of a hard mass, and lack of suspicious ultrasonographic findings.

Treatment

Considering the probable association between spironolactone and gynecomastia, spironolactone therapy was discontinued. The patient was continued on torsemide for edema management, with close monitoring of fluid status and electrolyte balance. Because the patient remained clinically stable after withdrawal of spironolactone, immediate initiation of alternative aldosterone antagonists was not considered necessary.

Eplerenone, a selective mineralocorticoid receptor antagonist associated with a lower incidence of gynecomastia, was considered as a potential alternative if edema worsened or became difficult to control during follow-up. Conservative management and regular monitoring were advised. Emollient therapy was prescribed for the hyperpigmented lesion on the foot. Surgical intervention was not indicated at this stage.

Outcome and follow-up

The patient returned for follow-up after four weeks in June 2024 while continuing torsemide 10 mg daily, metformin 500 mg, glimepiride 2 mg, and spironolactone 50 mg. Although modest breast enlargement remained, bilateral pedal edema was significantly alleviated, and breast pain had diminished. Serum electrolytes, liver and kidney function tests, and blood glucose showed very slight fluctuations. Estradiol decreased from 47 to 42 pg/mL and prolactin from 18.2 to 16.8 ng/mL. Spironolactone was then stopped because of the improvement in edema and persistent gynecomastia, whereas torsemide 10 mg daily was continued while fluid retention was monitored for recurrence. After four weeks, a follow-up was scheduled to evaluate fluid status and gynecomastia regression.

Learning points

Gynecomastia should always be considered in male patients receiving spironolactone, particularly in the presence of chronic liver disease. A detailed medication history and ultrasonography are important for excluding malignancy and identifying drug-induced causes.

## Discussion

The hallmarks of gynecomastia include breast enlargement, pain, and tenderness resulting from hormonal imbalance affecting glandular breast tissue [1]. Chronic liver disease is an important predisposing factor because impaired hepatic metabolism alters estrogen clearance, increases peripheral aromatization of androgens to estrogens, elevates sex hormone-binding globulin levels, and reduces free testosterone concentrations, thereby promoting glandular breast tissue proliferation [2]. Drug-induced gynecomastia has been associated with several medications, including spironolactone and digoxin [3]. Gynecomastia may occur physiologically or secondary to systemic disorders, endocrine abnormalities, or medications.

Spironolactone-induced gynecomastia occurs through multiple mechanisms, including inhibition of testosterone and dihydrotestosterone binding to androgen receptors, suppression of androgen receptor activity, inhibition of testosterone synthesis, and enhancement of peripheral conversion of testosterone to estradiol. Gynecomastia associated with spironolactone is often bilateral and related to both treatment duration and dosage [4]. Rose et al. demonstrated reduced serum testosterone levels with elevated estradiol concentrations among patients receiving spironolactone therapy, supporting hormonal imbalance as the underlying mechanism [5]. Engbaek et al. observed gynecomastia in 5.2% of patients receiving low-dose spironolactone therapy [6].

Spironolactone remains widely prescribed because of its effectiveness and availability in the management of edema and ascites associated with chronic liver disease [7]. Because eplerenone is more selective for mineralocorticoid receptors and has far less antiandrogenic effect than spironolactone, it might be a safer option. Due to the temporal correlation between drug exposure and the onset of symptoms, related hormonal abnormalities, and improvement after medication withdrawal, spironolactone was deemed the likely causal agent in this instance. The reaction was categorized as Level 2 (mild) according to the Modified Hartwig and Siegel severity assessment [8].

A published report has highlighted spironolactone-associated gynecomastia, particularly in patients receiving prolonged therapy or higher doses. Continued drug exposure may cause the condition to develop, and stopping treatment may allow it to progressively improve. Drug-induced gynecomastia should be identified as soon as possible because it can lead to psychological distress, cosmetic concerns, anxiety about cancer, and problems with medication adherence. For patients on long-term spironolactone therapy, it is crucial to carefully review their medication history, especially if they have underlying chronic liver disease and related endocrine disorders [9].

This case further emphasizes the importance of pharmacovigilance and adverse drug reaction reporting in clinical practice. It may be possible to reduce morbidity and improve patient compliance by identifying probable offending substances and considering alternative treatments such as eplerenone. A potential adverse drug reaction was indicated by a score of 6 obtained during causality assessment using the Naranjo Adverse Drug Reaction Probability Scale [10]. Given the clinical rationale for spironolactone therapy and the recognized but relatively common nature of this side effect, a preventability assessment using the Schumock and Thornton criteria indicated that the reaction was not certainly preventable. When patients present with bilateral breast enlargement during spironolactone therapy, clinicians should maintain a high index of suspicion for medication-induced endocrine side effects [11].

The link between spironolactone therapy and gynecomastia is further supported by additional research, especially when higher doses or longer treatment durations are used [12]. Evidence at the case level, such as a documented case of unilateral gynecomastia developing in a hypertensive patient after 12 months of spironolactone therapy, shows that this side effect is not exclusive to bilateral presentations and can occur after prolonged exposure, even at standard dosages [13]. All of these findings point to the necessity of ongoing clinical surveillance for gynecomastia in patients undergoing long-term or high-dose spironolactone therapy.

## Conclusions

This case demonstrates a clinically relevant instance of bilateral gynecomastia in a patient receiving long-term spironolactone therapy for chronic liver disease. Gynecomastia may have a complex etiology in this situation, with spironolactone having an additional antiandrogenic effect and the underlying liver disease potentially contributing to hormonal imbalance. When assessing gynecomastia in patients with chronic liver disease, it is crucial to consider long-term medication exposure, as demonstrated by the development of breast discomfort and enlargement despite ongoing low-dose spironolactone therapy. Individualized treatment may be guided by careful clinical evaluation, appropriate exclusion of alternative causes, and regular reevaluation of the ongoing need for spironolactone.
